# Blood pressure variability and blood pressure rhythm phenotypes in children with severe encephalitis complicated by paroxysmal sympathetic hyperactivity

**DOI:** 10.3389/fphar.2026.1906876

**Published:** 2026-08-12

**Authors:** Yufan Yang, Hengyun He, Wang Chen, Xinping Zhang, Jiyan Zhang, Jiaotian Huang, Xiulan Lu, Zhenghui Xiao

**Affiliations:** 1 Department of Intensive Care Unit, The School of Pediatrics, Hengyang Medical School, University of South China (Hunan Children’s Hospital), Changsha, Hunan, China; 2 Department of Intensive Care Unit, The affiliated Children’s Hospital of Xiangya School of Medicine, Central South University (Hunan Children’s Hospital), Changsha, Hunan, China; 3 Respiratory Department, The School of Pediatrics, Hengyang Medical School, University of South China (Hunan Children’s Hospital), Changsha, Hunan, China; 4 Respiratory Department, The affiliated Children’s Hospital of Xiangya School of Medicine, Central South University (Hunan Children’s Hospital), Changsha, Hunan, China

**Keywords:** blood pressure rhythm phenotype, blood pressure variability, children, paroxysmal sympathetic hyperactivity, severe encephalitis

## Abstract

**Objective:**

This study aimed to assess the effects of comorbid paroxysmal sympathetic hyperactivity (PSH) on disease severity, resource utilization, and the prognosis, thereby providing a basis for the identification and management of severe encephalitis with PSH in children.

**Methods:**

A retrospective analysis was conducted on the general clinical data, PSH-related manifestations, and resource utilization of children with severe encephalitis complicated by PSH who were admitted to the Pediatric Intensive Care Unit (PICU) of Hunan Children’s Hospital from January 2020 to December 2025.

**Results:**

A total of 28 children with severe encephalitis complicated by PSH were included. The distribution of blood pressure rhythm phenotypes was as follows: a nondipper pattern in 16 patients (57.1%) and a reverse-dipper pattern in 12 patients (42.9%), with no dipper or extreme-dipper patterns observed. A poor prognosis was observed in 16 patients (57.1%), including 5 fatalities. Furthermore, the PSH group had a significantly longer PICU length of stay, an increased duration of ventilator-assisted ventilation, a higher rate of mechanical ventilation use, and greater total hospitalization costs (all *P* < 0.001). Within the PSH-complicated group, the subgroup with a poor prognosis demonstrated a significantly higher coefficient of variation for diastolic blood pressure (*P* = 0.030) and a significantly lower GCS score at admission (*P* = 0.011).

**Conclusion:**

The majority of children with severe encephalitis complicated by PSH exhibited a nondipper or reverse-dipper blood pressure rhythm phenotype. Diastolic blood pressure variability and the GCS score at admission were associated with the prognosis. Compared with those in the control group, the children in the PSH-complicated group were older, experienced prolonged PICU stays, required more mechanical ventilation, and incurred significantly higher hospitalization costs.

## 1 Introduction

Severe encephalitis in children is a critical neurological emergency that poses a serious threat to their lives and health. It is often accompanied by multisystem dysfunction and is associated with high risks of disability and mortality. Various complications frequently arise during its course, including increased intracranial pressure, status epilepticus, and autonomic dysfunction (Palmas and Duke, 2023).

Among these complications, paroxysmal sympathetic hyperactivity (PSH) is a common complication following central nervous system injury. Its primary manifestations involve symptoms of sympathetic overactivity, such as fever, tachycardia, hypertension, sweating, and increased muscle tone (Baguley et al., 2014). The nomenclature and understanding of this syndrome have undergone a long evolution, having been previously described with various terms. In 2014, an international expert group, through a consensus conference, proposed “PSH” as the unified term to standardize related research and clinical practice (Baguley et al., 2014). Currently, the clinical presentation of PSH lacks specificity and is easily confused with conditions such as infection, seizures, or drug withdrawal, leading to frequent underdiagnosis and misdiagnosis. Furthermore, no universally accepted treatment protocol is available. Management often relies on empirical medication, resulting in variable efficacy. Some inappropriate interventions may even exacerbate brain injury or adversely affect prognosis (Althaqafi et al., 2025). The pathogenesis of PSH has not yet been fully elucidated. Existing research suggests that its mechanism involves structural damage disrupting the connections between higher cortical inhibitory brain regions and the brainstem or spinal/sympathetic nerve centers (Scott and Rabinstein, 2020). The underlying pathophysiology of PSH remains elusive, and the lack of objective physiological assessment tools makes the early identification and evaluation of the severity of PSH challenging.

Increased paroxysmal blood pressure is a typical manifestation of PSH, reflecting the sympathetic excitatory state and rhythmic changes in vascular tone regulation. Previous studies have focused primarily on peak or average blood pressure levels during episodes, paying insufficient attention to blood pressure variability and circadian patterns. The coefficient of variation for blood pressure can be used to quantitatively assess the degree of blood pressure fluctuation, while blood pressure rhythm phenotypes, such as nondipper and reverse-dipper patterns, can reveal disturbances in autonomic circadian control. Although autonomic dysfunction is important in various neurological diseases, systematic research on the blood pressure rhythm characteristics of pediatric patients with PSH and their clinical effects is lacking. Studies specifically reporting the relationship between PSH and blood pressure rhythm phenotypes are currently unavailable.

Therefore, this study focuses on children with severe encephalitis complicated by PSH. Based on 24-h invasive blood pressure monitoring data, the coefficients of variation for systolic and diastolic blood pressure were analyzed, and different blood pressure rhythm phenotypes were identified. The aim is to reveal objective patterns of sympathetic rhythm abnormalities in pediatric patients with PSH through quantifiable hemodynamic features, thereby providing new insights and evidence for the early identification, stratification of disease severity, and individualized management of PSH.

## 2 Subjects and methods

### 2.1 Study subjects

A total of 72 children admitted to the PICU of Hunan Children’s Hospital between January 2020 and December 2025 with a diagnosis of severe encephalitis and complete clinical data were included in this study. Based on the presence or absence of comorbid PSH, the children were retrospectively divided into two groups for analysis: the Comorbid Group (n = 28) and the Control Group (n = 44). Within the Comorbid Group, the subjects were further subdivided according to discharge and follow-up outcomes into a favorable prognosis subgroup (n = 12) and an unfavorable prognosis subgroup (n = 16). This manuscript has obtained human research ethics approval from Ethics Committee of Hunan Children’s Hospital on April 2026 in accordance with the Declaration of Helsinki, approval number: HCHLL-2026–88. We have received written informed patient consent to perform this study.

A favorable prognosis was defined as the complete resolution of clinical symptoms following active treatment, recovery of consciousness and neurological function to normal levels, absence of significant sequelae or functional impairment, and no requirement for long-term rehabilitation therapy during the follow-up period.

An unfavorable prognosis was defined as follows: in-hospital mortality or the presence of significant neurological deficits at discharge requiring long-term rehabilitation therapy (≥6 months), recurrent hospitalizations, or an inability to resume activities of daily living.

The prognostic assessment was based on discharge records and follow-up data (follow-up period: 6–12 months after discharge) and was jointly determined by two neurologists specializing in the field.

The results of the analysis of blood pressure rhythm phenotypes revealed that the nocturnal blood pressure diurnal patterns of all 28 children in the Comorbid Group were of the nondipper or reverse-dipper type. Therefore, they were further divided into a nondipper group (n = 16) and a reverse-dipper group (n = 12) for comparative analysis.

#### 2.1.1 Inclusion criteria for the comorbid group

Children were eligible for inclusion if they were aged 0–18 years, regardless of sex; were hospitalized with a definitive diagnosis of severe encephalitis complicated by PSH; and had complete medical records and blood pressure monitoring data, including invasive blood pressure monitoring for at least 24 h.

#### 2.1.2 Exclusion criteria for the comorbid group

Children were excluded if they had factors that might affect autonomic function or blood pressure variability, including primary or secondary hypertension, a history of cranial surgery, or other acute central nervous system or systemic diseases that could present with manifestations similar to PSH. Patients with incomplete medical records or missing key clinical data were also excluded.

#### 2.1.3 Control group

The Control Group consisted of hospitalized children who were definitively diagnosed with severe encephalitis without comorbid PSH and admitted during the same period. They had complete clinical data, their age range matched that of the Comorbid Group, and the exclusion criteria applied were the same as those for the Comorbid Group.

### 2.2 Data collection

The data recorded for each child included sex, age, the Glasgow Coma Scale (GCS) score at admission, vital signs such as body temperature, respiratory rate, heart rate, 24-h invasive blood pressure monitoring results, duration of mechanical ventilation, PICU length of stay, treatment costs, and clinical outcome.

### 2.3 PSH diagnostic criteria

The diagnosis of PSH used in this study was based on the Paroxysmal Sympathetic Hyperactivity Assessment Measure (PSH-AM) proposed by Baguley et al., in 2014 (Baguley et al., 2014). This scale consists of two subscales: the Clinical Features Scale (CFS) and the Diagnosis Likelihood Tool (DLT). The CFS is used to assess specific clinical manifestations of PSH, with each item scored from 0 to 3 points. The DLT is used to judge the specificity of the diagnosis and comprises a total of 11 diagnostic items. The scores from both subscales are summed to obtain a total score, which is used to determine the likelihood of PSH: a total score < 8 indicates “unlikely,” 8–16 indicates “possible,” and ≥17 indicates “highly likely”. In this study, a PSH-AM total score ≥ 8 was defined as the diagnostic criterion for PSH.

### 2.4 Diagnostic criteria for pediatric hypertension

This study adopted internationally authoritative guidelines to define diagnostic criteria for pediatric hypertension, ensuring a consistent determination of the hypertensive status throughout the research. The specific criteria are based on the American Academy of Pediatrics (AAP) 2017 Clinical Practice Guidelines for the Screening and Management of High Blood Pressure in Children and Adolescents (Flynn et al., 2017), as follows.

1. For children under 13 years of age, blood pressure levels must be evaluated against reference values corresponding to their age, sex, and height percentile. A single measurement or repeated measurements showing a systolic blood pressure (SBP) or diastolic blood pressure (DBP) ≥ the 95th percentile for age, sex, and height can be considered indicators of hypertension.
2. Absolute blood pressure values may be used for adolescents aged 13 years and older. For example, an SBP ≥ 130 mmHg or a DBP ≥ 80 mmHg serves as the reference threshold for the diagnosis of hypertension.

Given that the scoring thresholds for blood pressure-related items in the PSH-AM scale are based on reference ranges for adult and the population in this study consists of pediatric patients, directly applying adult blood pressure standards may lead to a misjudgment of a sympathetic activation-related blood pressure elevation. Elevated blood pressure was redefined in this study in accordance with the 2017 American Academy of Pediatrics (AAP) Clinical Practice Guidelines for the Screening and Management of High Blood Pressure in Children and Adolescents to ensure age-specific accuracy in the assessment. Based on these guidelines, the adult standards corresponding to the scoring items for elevated blood pressure in the PSH-AM scale were replaced with the aforementioned pediatric blood pressure criteria while the original scoring structure of the scale was retained. This adaptation was made to ensure the applicability and scoring consistency of the scale within the pediatric population. However, this modified pediatric version has not yet undergone formal validation.

### 2.5 Classification of blood pressure rhythms

**FIGURE 1:**
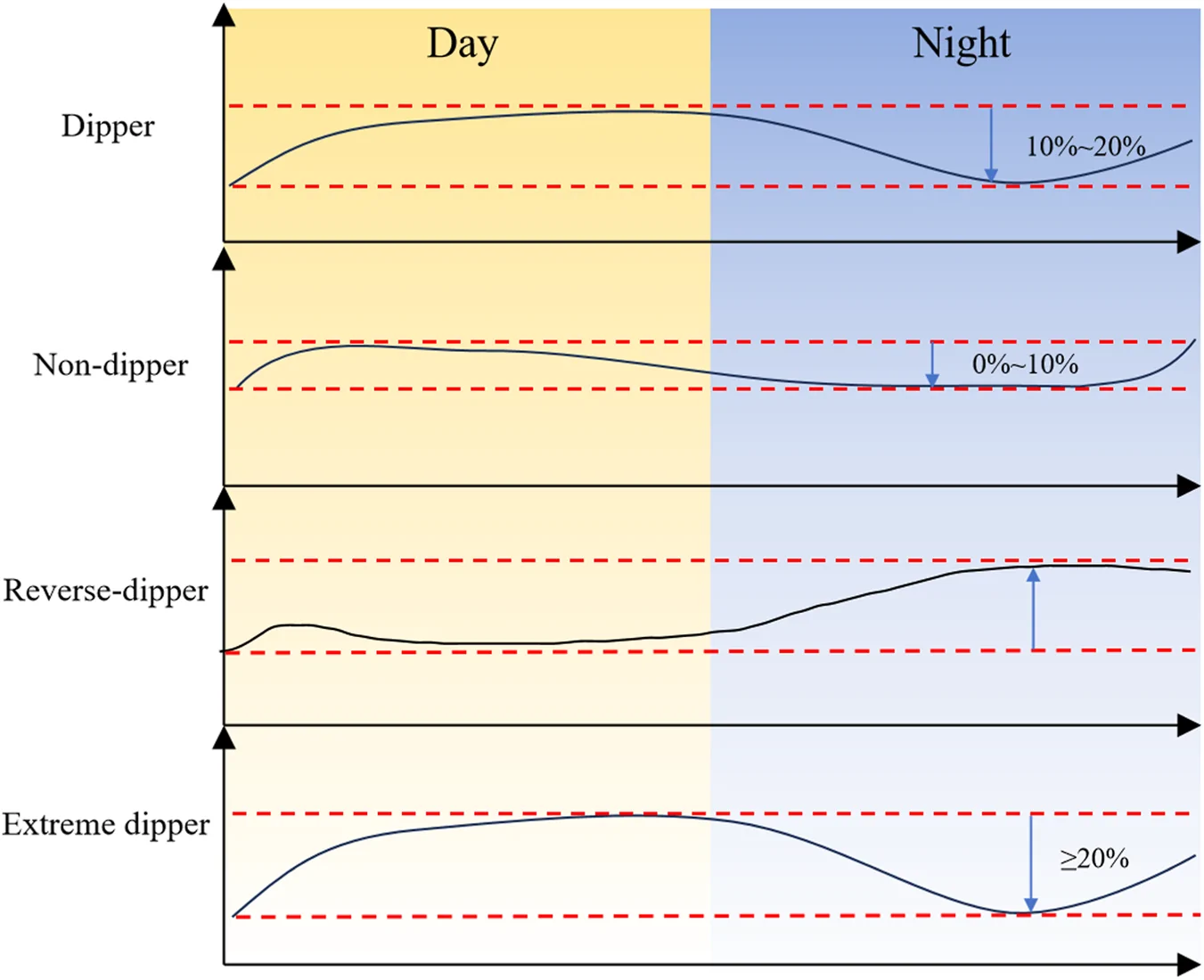
Circadian blood pressure rhythm phenotypes in 24-h invasive blood pressure monitoring. Based on the decline in nighttime average systolic blood pressure (SBP) compared to daytime average SBP, circadian blood pressure rhythms are classified into four types: **(A)** Dipper blood pressure: nighttime decline rate ranges between 10% and 20%; **(B)** Non-dipper blood pressure: nighttime decline rate ranges between 0% and 10%; **(C)** Reverse dipper blood pressure: nighttime average blood pressure is higher than daytime average blood pressure; **(D)** Extreme dipper blood pressure: nighttime average blood pressure is significantly lower than daytime average blood pressure (nighttime decline rate ≥20%).

Under normal conditions, a significant difference exists between daytime and nighttime blood pressure values. A physiological decrease in nighttime blood pressure is considered a normal circadian pattern, and individuals exhibiting this characteristic decrease in nocturnal blood pressure are typically referred to as having a dipper blood pressure profile. Based on the percentage decrease in the average nighttime systolic blood pressure (SBP) compared with the average daytime SBP, the circadian blood pressure rhythm can be classified into the following four types (Figure 1) (Williams et al., 2018).

1. Dipper—nocturnal blood pressure decreases between 10% and 20%.
2. Nondipper—nocturnal blood pressure decreases between 0% and 10%.
3. Reverse dipper (or Risor)—the average nighttime blood pressure is higher than the average daytime blood pressure (nocturnal decrease rate < 0%).
4. Extreme dipper (or overdipper)—the average nighttime blood pressure is markedly lower than the average daytime blood pressure (nocturnal decrease rate ≥ 20%).

The nocturnal dip rate was calculated using the following formula:

Percent dip (%) = [(Daytime Mean BP–Nighttime Mean BP)/Daytime Mean BP] × 100%

For circadian rhythm analysis, the 24-h period was divided into daytime and nighttime. Daytime was defined as 06:00–18:00, and nighttime was defined as 18:00–06:00 on the following day.

#### 2.5.1 Blood pressure monitoring and variability assessment

Blood pressure measurements were obtained through continuous bedside monitoring using an invasive arterial catheter placed as part of routine intensive care management. The arterial pressure waveform was continuously recorded, and hourly SBP and diastolic blood pressure (DBP) values were extracted for subsequent analysis. In addition, because 24-h invasive blood pressure monitoring was performed during the active phase of PSH according to clinical circumstances, variability in the timing of assessment may have influenced the evaluation of blood pressure variability and blood pressure rhythm phenotypes.

### 2.6 Diagnostic criteria for severe encephalitis

This study employed established international consensus and authoritative diagnostic frameworks for encephalitis and severe encephalitis to confirm cases among enrolled children. The specific criteria are as follows (Venkatesan et al., 2013).

1. Major Diagnostic Criterion—the presence of an altered mental status (e.g., confusion, lethargy, disorientation, etc.) persisting for ≥ 24 h, with other explanatory causes excluded.
2. Minor Criteria (the fulfillment of ≥2 items is required for a possible diagnosis of encephalitis, and ≥3 items for a confirmed diagnosis)
3. Fever ≥ 38 °C within 72 h before or after illness onset.
4. Generalized or focal seizures that cannot be fully attributed to a pre-existing seizure disorder.
5. New-onset focal neurological signs.
6. Cerebrospinal fluid (CSF) pleocytosis (white blood cell count > 5 cells/mm^3^).
7. Neuroimaging findings showing brain parenchymal abnormalities suggestive of encephalitis, which are newly identified or acute in onset compared with prior examinations.
8. Electroencephalogram (EEG) abnormalities are consistent with encephalitis and are not attributable to other causes.
9. Based on the above criteria, a diagnosis of encephalitis is made if the major criterion is met along with ≥3 minor criteria. Cases can be classified as “severe encephalitis” if they are accompanied by admission to the PICU, a requirement for mechanical ventilation, profound depression of consciousness, rapid disease progression, or the presence of multiorgan dysfunction.

### 2.7 Statistical methods

All the data were statistically analyzed using SPSS version 27.0. Measurement data conforming to a normal distribution are presented as the means ± standard deviations ( $\bar{x}$ ± s), whereas those not conforming to a normal distribution are described as the medians (interquartile ranges), M (IQRs). For continuous data with a normal distribution, independent sample t tests were used for between-group comparisons; for data not conforming to a normal distribution, the Mann‒Whitney U test was employed. Count data are presented as rates or constituent ratios (%), with between-group comparisons performed using the χ^2^ test or Fisher’s exact probability test. The significance level was set at α = 0.05, with *P* < 0.05 considered to indicate statistical significance.

## 3 Results

### 3.1 Baseline clinical characteristics of children with PSH

A total of 28 children who were diagnosed with severe encephalitis complicated by PSH were included in this study, including 19 males (67.9%) and 9 females (32.1%). The median age of the children was 8.20 years (IQR: 5.08, 12.58). Regarding the clinical manifestations, the majority of the children presented with fever (26 cases; 92.9%), tachycardia (27 cases; 96.4%), and sweating (26 cases; 92.9%). Tachypnea was observed in 22 patients (78.6%). All the children exhibited hypertension (28 cases, 100%), and 8 children (28.6%) presented with increased muscle tone. In terms of etiology, 13 patients had infectious encephalitis (46.4%), while 15 patients had noninfectious encephalitis (53.6%). Blood pressure rhythm phenotyping revealed that all the children exhibited either a nondipper or reverse-dipper pattern: 16 patients (57.1%) had a nondipper pattern, and 12 patients (42.9%) had a reverse-dipper pattern. Blood pressure monitoring results showed a systolic blood pressure coefficient of variation of 9.08% ± 3.18% and a diastolic blood pressure coefficient of variation of 11.41% ± 3.92%. The time from symptom onset to a definitive PSH diagnosis was 8.00 days (IQR: 5.25, 12.75). The GCS score upon admission to the PICU was 6.71 ± 3.50. In terms of the prognosis, 12 patients (42.9%) had favorable outcomes, while 16 patients (57.1%) had unfavorable outcomes, including 5 fatalities (Table 1).

**TABLE 1:** Baseline clinical characteristics of children with severe encephalitis complicated by PSH.

Item	Value	Item	Value
Number of patients (n)	28	Age, M (IQR)	8.20 (5.08, 12.58)
Sex [n (%)]	​	Prognosis [n (%)]	​
Male	19 (67.9%)	Favorable	12 (42.9%)
Female	9 (32.1%)	Unfavorable	16 (57.1%)
Fever [n (%)]	​	Hypertension [n (%)]	​
Present	26 (92.9%)	Present	28 (100%)
Absent	2 (7.1%)	Absent	0 (0%)
Tachypnea [n (%)]	​	Tachycardia [n (%)]	​
Present	22 (78.6%)	Present	27 (96.4%)
Absent	6 (21.4%)	Absent	1 (3.6%)
Sweating [n (%)]	​	Increased muscle tone [n (%)]	​
Present	26 (92.9%)	Present	8 (28.6%)
Absent	2 (7.1%)	Absent	20 (71.4%)
Etiology [n (%)]	​	Blood pressure rhythm phenotype [n (%)]	​
Infectious	13 (46.4%)	Nondipper	16 (57.1%)
Noninfectious	15 (53.6%)	Reverse-dipper	12 (42.9%)
Coefficient of variation for systolic blood pressure (%, $\bar{x}$ ± s)	9.08 ± 3.18	Coefficient of variation for diastolic blood pressure (%, $\bar{x}$ ± s)	11.41 ± 3.92
Time from symptom onset to PSH diagnosis (days), M (IQR)	8.00 (5.25, 12.75)	GCS score on admission ( $\bar{x}$ ± s)	6.71 ± 3.50

### 3.2 Comparison of hospitalization characteristics between the comorbid group and the control group

Among the 72 children with severe encephalitis included in the study, 28 were included in the PSH-comorbid group and 44 were included in the control group. In terms of the sex composition, the comorbid group consisted of 19 males (67.9%) and 9 females (32.1%), whereas the control group included 20 males (45.4%) and 24 females (54.5%). No statistically significant difference in the sex distribution was observed between the two groups. The median age of patients in the PSH-comorbid group was 8.20 years (IQR: 5.08, 12.58), which was significantly older than the 5.70 years (IQR: 2.30, 7.48) of patients in the control group (*P* = 0.014). In terms of the etiology, the comorbid group included 13 patients with an infectious etiology (46.4%) and 15 with a noninfectious etiology (53.6%) compared with 31 patients with an infectious etiology (70.5%) and 13 with a noninfectious etiology (29.5%) in the control group, with no statistically significant difference observed.

The median PICU length of stay of patients in the PSH-comorbid group was 15.00 days (IQR: 9.50, 28.00), which was significantly longer than the 10.00 days (IQR: 7.00, 14.00) for patients in the control group (*P* = 0.004). The duration of assisted ventilation was also significantly longer in the comorbid group, with a median of 7.50 days (IQR: 3.25, 13.50) compared with 0.00 days (IQR: 0.00, 4.00) in the control group (*P* < 0.001). The mechanical ventilation utilization rate was significantly higher in the PSH-comorbid group (78.6%) than in the control group (36.4%) (*P* < 0.001). The median hospitalization cost was significantly higher in the PSH-comorbid group (83,185.47 Chinese yuan; IQR: 56,486.29, 132,408.05) than in the control group (39,335.61 Chinese yuan; IQR: 25,812.29, 55,726.63) (*P* < 0.001). Furthermore, the proportion of patients with an unfavorable prognosis was significantly higher in the comorbid group (57.1%) than in the control group (27.3%) (*P* = 0.014). Sedative use was significantly more frequent in the PSH-comorbid group, with 25 patients (89.3%) receiving sedation, compared with 25 patients (56.8%) in the control group (*P* = 0.004). Vasopressor therapy was also used more frequently in the PSH-comorbid group, with 8 patients (28.6%) requiring vasopressors compared with 2 patients (4.5%) in the control group (*P* = 0.011) (Table 2).

**TABLE 2:** Comparison of the clinical characteristics and resource utilization between the comorbid group and the control group.

Item	Comorbid group	Control group	Statistic	*P*
Number of patients	28	44	​	​
Sex	​	​	χ^2^ = 3.459	0.090
Male	19 (67.9%)	20 (45.5%)	​	​
Female	9 (32.1%)	24 (54.5%)	​	​
Age, M (IQR)	8.20 (5.08, 12.58)	5.70 (2.30, 7.48)	Z = −2.461	0.014*
Etiology	​	​	χ^2^ = 4.156	0.051
Infectious	13 (46.4%)	31 (70.5%)	​	​
Noninfectious	15 (53.6%)	13 (29.5%)	​	​
PICU length of stay, M (IQR)	15.00 (9.50, 28.00)	10.00 (7.00, 14.00)	Z = −2.844	0.004*
Duration of ventilator-assisted ventilation, M (IQR)	7.50 (3.25, 13.50)	0.00 (0.00, 4.00)	Z = −4.277	<0.001*
Mechanical ventilation	​	​	χ^2^ = 12.231	<0.001*
Yes	22 (78.6%)	16 (36.4%)	​	​
No	6 (21.4%)	28 (63.6%)	​	​
Hospitalization cost, M (IQR)	83185.47 (56486.29, 132408.05)	39335.61 (25812.29, 55726.63)	Z = −4.990	<0.001*
Sedation	​	​	χ^2^ = 8.501	0.004*
Yes	25 (89.3%)	25 (56.8%)	​	​
No	3 (10.7%)	19 (43.2%)	​	​
Vasopressor	​	​	Fisher	0.011*
Yes	8 (28.6%)	2 (4.5%)	​	​
No	20 (71.4%)	42 (95.5%)	​	​
Prognosis	​	​	χ^2^ = 6.424	0.014*
Favorable	12 (42.9%)	32 (72.7%)	​	​
Unfavorable	16 (57.1%)	12 (27.3%)	​	​

A statistically significant difference (*P* < 0.05) is indicated by the asterisk (*).

### 3.3 Comparison of different prognostic outcomes in children with severe encephalitis complicated by PSH

With respect to the demographic characteristics, the favorable prognosis group included 12 children, with 9 males (75.0%) and 3 females (25.0%). The unfavorable prognosis group included 16 children, with 10 males (62.5%) and 6 females (37.5%). The median ages were 8.45 years (IQR: 6.03, 12.33) and 6.90 years (IQR: 2.80, 12.75), respectively, with no statistically significant difference between the two groups. In terms of blood pressure rhythm phenotypes, the favorable prognosis group consisted of 9 patients (75.0%) with the non-dipper pattern and 3 patients (25.0%) with the reverse-dipper pattern. The unfavorable prognosis group consisted of 7 patients (43.8%) with the nondipper pattern and 9 patients (56.3%) with the reverse-dipper pattern. The coefficient of variation for systolic blood pressure was 7.87% ± 2.75% in the favorable prognosis group and 9.98% ± 3.25% in the unfavorable prognosis group; this difference was not statistically significant. The coefficient of variation for diastolic blood pressure was 9.59% ± 3.03% in the favorable prognosis group and 12.78% ± 4.04% in the unfavorable prognosis group, a difference that was statistically significant (*P* = 0.030). In terms of the etiology, the favorable prognosis group included 4 patients with an infectious etiology (33.3%) and 8 patients with a noninfectious etiology (66.7%), whereas the unfavorable prognosis group included 9 patients with an infectious etiology (56.3%) and 7 patients with a noninfectious etiology (43.8%). The difference in etiology between the two groups was not statistically significant. The time from symptom onset to the PSH diagnosis was 9.50 days (IQR: 7.25, 13.75) in the favorable prognosis group and 7.00 days (IQR: 5.00, 12.75) in the unfavorable prognosis group, with no statistically significant difference. Regarding treatment-related factors, sedation was used in 11 patients (91.7%) in the favorable prognosis group and 14 patients (87.5%) in the unfavorable prognosis group (*P* = 1.000). Vasopressor use was observed in 1 patient (8.3%) in the favorable prognosis group and 7 patients (43.7%) in the unfavorable prognosis group (*P* = 0.088). The mean GCS scores were 8.58 ± 3.26 and 5.31 ± 3.05 for the two groups, respectively, which were significantly different (*P* = 0.011) (Table 3).

**TABLE 3:** Comparison of different prognostic outcomes in children with severe encephalitis complicated by PSH.

Item	Favorable prognosis group	Unfavorable prognosis group	Statistic	*P*
Sex (Male/Female)	​	​	Fisher	0.687
Male	9 (75.0%)	10 (62.5%)	​	​
Female	3 (25.0%)	6 (37.5%)	​	​
Age, M (IQR)	8.45 (6.03, 12.33)	6.90 (2.80, 12.75)	Z = −0.488	0.626
Blood pressure rhythm phenotype	​	​	χ^2^ = 2.734	0.136
Nondipper	9 (75.0%)	7 (43.8%)	​	​
Reverse-dipper	3 (25.0%)	9 (56.3%)	​	​
Coefficient of variation for systolic BP (%, $\bar{x}$ ± s)	7.87 ± 2.75	9.98 ± 3.25	t = −1.812	0.082
Coefficient of variation for diastolic BP (%, $\bar{x}$ ± s)	9.59 ± 3.03	12.78 ± 4.04	t = −2.293	0.030^*^
Etiology	​	​	χ^2^ = 1.448	0.276
Infectious	4 (33.3%)	9 (56.3%)	​	​
Noninfectious	8 (66.7%)	7 (43.8%)	​	​
Time from symptom onset to PSH diagnosis, M (IQR)	9.50 (7.25, 13.75)	7.00 (5.00, 12.75)	Z = −1.492	0.136
GCS score at admission ( $\bar{x}$ ± s)	8.58 ± 3.26	5.31 ± 3.05	t = 2.728	0.011^*^
Sedation	​	​	Fisher	1.000
Yes	11 (91.7%)	14 (87.5%)	​	​
No	1 (8.3%)	2 (12.5%)	​	​
Vasopressor	​	​	Fisher	0.088
Yes	1 (8.3%)	7 (43.7%)	​	​
No	11 (91.7%)	9 (56.3%)	​	​

**FIGURE 2:**
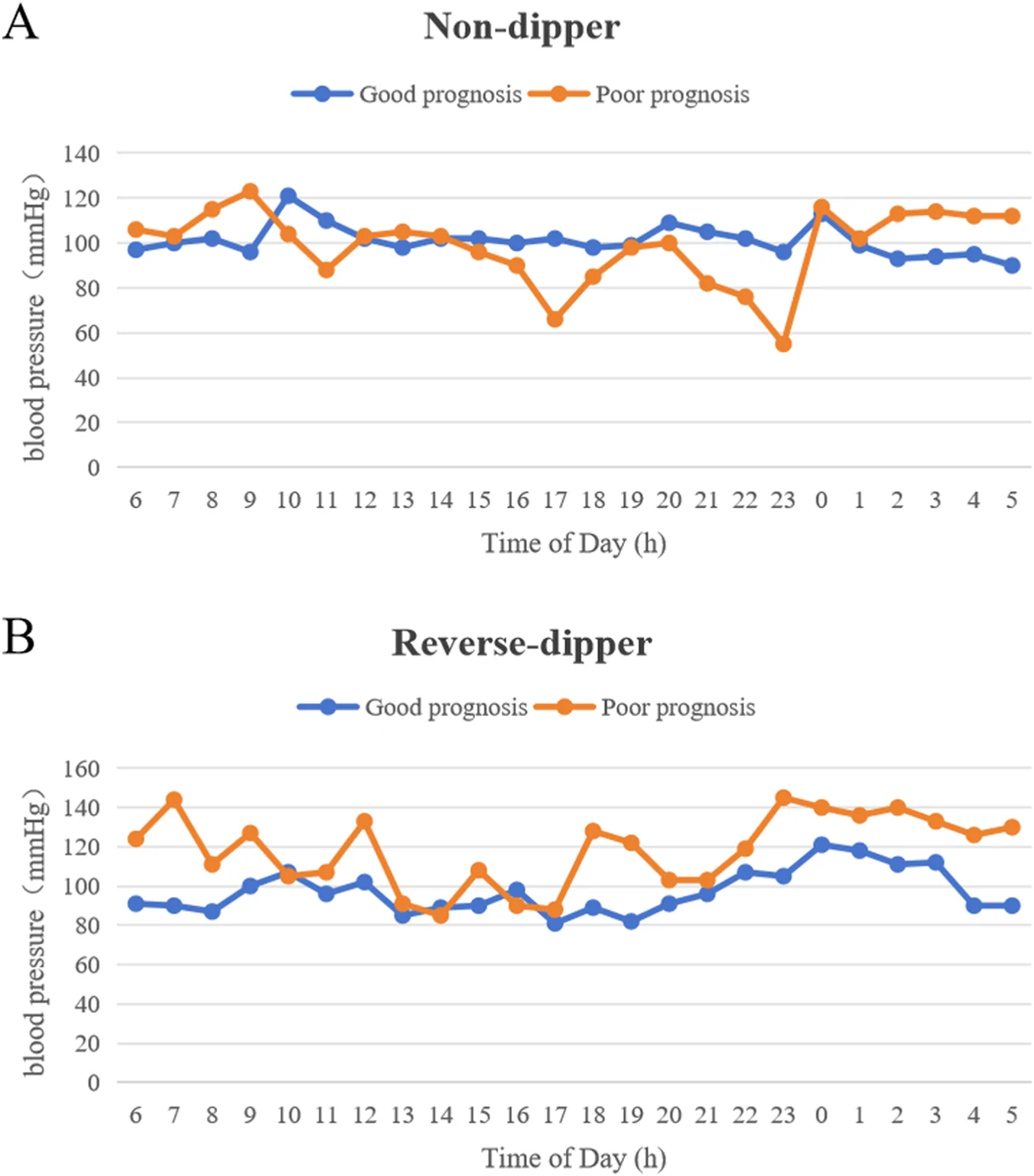
The relationship between 24-h blood pressure variability and prognosis in children with different blood pressure rhythm phenotypes. The curves represent individual patients’ systolic blood pressure trajectories over 24 h, with each pair of curves representing two representative patients from the corresponding comparison groups. **(A)** Non-dipper blood pressure children: The poor-prognosis group exhibited more pronounced 24-h blood pressure fluctuations and greater amplitude, while the favourable prognosis group showed relatively stable blood pressure curves. **(B)** Reverse-dipper blood pressure children: The poor-prognosis group also displayed a wider range of blood pressure fluctuations, whereas the favourable prognosis group demonstrated smaller variations and more gradual fluctuations.

Among children with the nondipper pattern, those with an unfavorable prognosis exhibited more pronounced and larger-amplitude fluctuations in 24-h systolic blood pressure, whereas those with a favorable prognosis displayed relatively stable blood pressure curves (Figure 2A). Among children with the reverse-dipper pattern, those with an unfavorable prognosis still displayed a greater range of blood pressure fluctuations, whereas those with a favorable prognosis showed more gradual blood pressure changes (Figure 2B).

### 3.4 Comparison of different blood pressure rhythm phenotypes in children with severe encephalitis complicated by PSH

With respect to the sex distribution, the nondipper group included 16 children, with 11 males (68.8%) and 5 females (31.3%). The reverse-dipper group included 12 children, with 8 males (66.7%) and 4 females (33.3%). The median ages were 7.75 years (IQR: 5.08, 11.03) for the nondipper group and 11.35 years (IQR: 4.75, 12.68) for the reverse-dipper group, with no statistically significant difference between the two groups. In terms of blood pressure variability, the coefficient of variation for systolic blood pressure was 8.79% ± 3.38% in the nondipper group and 9.46% ± 2.98% in the reverse-dipper group; this difference was not statistically significant. The coefficients of variation for diastolic blood pressure were 11.08% ± 3.37% and 11.85% ± 4.69% for the two groups, respectively, which were not significantly different. In terms of the etiology, the composition was similar between the two groups. The nondipper group included 8 patients with an infectious etiology (50.0%) and 8 with a noninfectious etiology (50.0%). The reverse-dipper group included 5 patients with an infectious etiology (41.7%) and 7 with a noninfectious etiology (58.3%). The difference in etiology between the groups was not statistically significant. The mean time from symptom onset to the PSH diagnosis was 9.81 ± 4.83 days in the nondipper group and 8.50 ± 4.30 days in the reverse-dipper group, with no statistically significant difference. The mean GCS scores were 6.81 ± 3.62 and 6.58 ± 3.48 for the two groups, respectively, again showing no statistically significant difference. Regarding treatment-related factors, sedation was used in 15 patients (93.8%) in the nondipper group and 10 patients (83.3%) in the reverse-dipper group (*P* = 0.560). Vasopressor use was observed in 3 patients (18.7%) in the nondipper group and 5 patients (41.7%) in the reverse-dipper group (*P* = 0.231). In terms of the prognosis, the nondipper group included 9 patients (56.3%) with favorable outcomes and 7 patients (43.8%) with unfavorable outcomes. The reverse-dipper group included 3 patients (25.0%) with favorable outcomes and 9 patients (75.0%) with unfavorable outcomes. This difference was not statistically significant (Table 4).

**TABLE 4:** Comparison of different blood pressure rhythm phenotypes in children with severe encephalitis complicated by PSH.

Item	Nondipper group	Reverse-dipper group	Statistic	*P*
Sex	​	​	Fisher	1.000
Male	11 (68.8%)	8 (66.7%)	​	​
Female	5 (31.3%)	4 (33.3%)	​	​
Age, M (IQR)	7.75 (5.08, 11.03)	11.35 (4.75, 12.68)	Z = −0.790	0.430
Coefficient of variation for systolic BP (%, $\bar{x}$ ± s)	8.79 ± 3.38	9.46 ± 2.98	t = −0.548	0.588
Coefficient of variation for diastolic BP (%, $\bar{x}$ ± s)	11.08 ± 3.37	11.85 ± 4.69	t = −0.507	0.616
Etiology	​	​	χ^2^ = 0.191	0.718
Infectious	8 (50.0%)	5 (41.7%)	​	​
Noninfectious	8 (50.0%)	7 (58.3%)	​	​
Time from symptom onset to PSH diagnosis (days, $\bar{x}$ ± s)	9.81 ± 4.83	8.50 ± 4.30	t = 0.745	0.463
GCS score at admission ( $\bar{x}$ ± s)	6.81 ± 3.62	6.58 ± 3.48	t = 0.169	0.867
Prognosis	​	​	χ^2^ = 2.734	0.136
Favorable	9 (56.3%)	3 (25.0%)	​	​
Unfavorable	7 (43.8%)	9 (75.0%)	​	​
Sedation	​	​	Fisher	0.560
Yes	15 (93.8%)	10 (83.3%)	​	​
No	1 (6.2%)	2 (16.7%)	​	​
Vasopressor	​	​	Fisher	0.231
Yes	3 (18.7%)	5 (41.7%)	​	​
No	13 (81.3%)	7 (58.3%)	​	​

## 4 Discussion

While PSH is most commonly associated with traumatic brain injury, it can also occur in response to nontraumatic acute brain injuries, such as hypoxic–ischemic brain injury following cardiac arrest, intracranial hemorrhage, and certain autoimmune or infectious encephalitides (Hughes and Rabinstein, 2014; Kirk et al., 2012; Mittal et al., 2016). Notably, PSH following global hypoxia–ischemia or encephalitis appears to be more common in pediatric patients (Kirk et al., 2012; Meyfroidt et al., 2017). These findings suggest that PSH can manifest in various contexts of acute brain injury and that its occurrence may be influenced by factors such as the stage of neurological development, the type of injury, and its severity. In pediatric patients with acute brain injury, in addition to patients with traumatic brain injury and hypoxic–ischemic injury, severe encephalitis can also present with manifestations of paroxysmal sympathetic hyperactivity. This finding provides a theoretical basis for the investigation and analysis of children with severe encephalitis complicated by PSH in the present study.

PSH episodes are often paroxysmal, with external stimuli generally considered the most common triggers. These stimuli include common clinical procedures such as endotracheal suctioning, loud noise stimulation, position changes, and urinary retention. However, PSH is not always triggered externally; in some children, sympathetic storms can occur even in the absence of obvious stimuli. This result suggests that its underlying mechanism is not solely influenced by exogenous triggers but may more significantly reflect a state of high instability within the central autonomic regulatory system (Fernandez-Ortega et al., 2017). Previous research has also indicated a significant association between ambient temperature regulation and the occurrence of PSH. Lower environmental temperatures can increase the risk of sympathetic activation, whereas warming measures, such as increased blanket use, have also been linked to a higher probability of episodes (Letzkus et al., 2018a). These findings suggest that somatosensory input and temperature-related stimuli may increase peripheral afferent signaling, thereby exacerbating central sensitivity to sympathetic reflexes. The mechanism triggering PSH likely results from the combined effects of external stimuli and a damaged central regulatory network. Clinical management should aim to avoid unnecessary stimuli and strengthen the monitoring of temperature variations and the intensity of nursing procedures to mitigate the effects of potential precipitating factors on the disease course.

Among the 28 children with severe encephalitis complicated by PSH in this study, males constituted a relatively higher proportion, with a median age of approximately 8.2 years. These findings provide preliminary evidence that pediatric PSH may be more common among preschool- and school-aged children. The older age observed in the PSH group may partly reflect the age distribution of severe encephalitis complicated by PSH. However, because blood pressure regulation and circadian rhythm mature throughout childhood, the potential influence of age on BP variability cannot be completely excluded. Regarding the clinical manifestations, nearly all the children presented with fever, tachycardia, sweating, and hypertension, with tachypnea also being relatively common. These symptoms are generally consistent with the classic presentation of PSH. Some children also exhibited increased muscle tone, which is consistent with the reported proportions of muscle tone and postural abnormalities in previous literature (Hughes and Rabinstein, 2014). Notably, when increased muscle tone, convulsions, and impaired consciousness coexist, PSH may be misdiagnosed as a seizure episode in the clinic. Markedly increased muscle tone and abnormal postures are typically observed during the most severe episodes and can be mistaken for tonic seizures, with abnormal postures usually being symmetrical. In this study, the majority of children did not present with isolated seizures but rather with symptoms accompanying autonomic storms. This finding highlights the need for clinicians to maintain a high index of suspicion for PSH when managing children with severe encephalitis to avoid a misdiagnosis or missed diagnosis. A critical care study noted that the typical signs of PSH often peak around days 9–13 of the disease course (Alofisan et al., 2019). The temporal distribution observed in the present study is similar, providing preliminary support for the possibility that PSH may be more frequently recognized during the first to second weeks following severe brain injury. Clinically, this pattern has significant warning implications. During this critical window in the course of severe encephalitis in children, dynamic monitoring of vital sign fluctuations and heightened vigilance for characteristic PSH episodes are essential to prevent misdiagnosis and delayed intervention, thereby facilitating the timely initiation of targeted treatment.

In this study, children with severe encephalitis complicated by PSH displayed a significantly higher disease burden than children in the control group. This higher disease burden was evidenced by a prolonged PICU length of stay, a longer duration of assisted ventilation, a higher rate of mechanical ventilation use, increased hospitalization costs, and a higher proportion of unfavorable outcomes. This trend is consistent with findings from previous studies, which have indicated that PSH in children is associated with extended hospital stays, an increased need for mechanical ventilation, and greater overall disease severity (Agrwal et al., 2022; Letzkus et al., 2018b; Pozzi et al., 2017). However, the current evidence is insufficient to definitively establish whether PSH directly worsens the disease course or whether it primarily represents a clinical manifestation of the severity of the primary brain injury itself. Some studies propose that the persistent sympathetic overactivation induced by PSH can lead to hypermetabolism, an increased cardiopulmonary load, increased nutritional consumption, and an elevated risk of secondary infections, thereby increasing morbidity and prolonging the recovery time (SEO, 2023). Conversely, other studies suggest that the emergence of PSH is often linked to more severe neurological damage, impaired consciousness, and disruption of functional networks in the brain. Therefore, its association with unfavorable outcomes might be confounded by the factor “disease severity” (Agrwal et al., 2022). The higher frequency of sedative and vasopressor use in the PSH-comorbid group is consistent with the greater severity of illness and the increased need for intensive care support in these patients. Children with severe encephalitis complicated by PSH are more likely to require mechanical ventilation, close hemodynamic monitoring, and pharmacological support during the acute stage. Accordingly, the greater use of these therapies in the PSH-comorbid group is likely to reflect the complexity of clinical management rather than an isolated treatment characteristic.

A further comparison from an etiological perspective between the findings of this study and those reported in the previous literature reveals that PSH can occur across a spectrum of central nervous system diseases. For instance, in cohort studies of children after brain tumor resection, PSH is uncommon but it tends to be observed in younger patients with more invasive tumors near the ventricles or brainstem. These children also experience longer hospital stays, an increased need for cerebrospinal fluid shunting, and overall poorer prognoses (Bouchal et al., 2023). Furthermore, more than one-quarter of children with anti-NMDAR encephalitis develop PSH during the acute phase. This condition is often accompanied by deeper and more prolonged impairment of consciousness, more severe neurological deficits, and significantly extended hospital stays, demonstrating a clear aggravating effect of PSH on the disease course (Feng et al., 2021). In pediatric patients with tuberculous meningitis, PSH also frequently emerges as a late complication during disease progression, with the risk being particularly elevated when complicated by hydrocephalus (Jauhari et al., 2024). The consistent trends observed across different disease entities suggest that the emergence of PSH may represent a common phenotype of central autonomic dysregulation and is closely associated with a more severe disease trajectory.

In this study, children with an unfavorable prognosis exhibited greater diastolic blood pressure variability, while no significant difference was observed in systolic blood pressure variability. The increased diastolic blood pressure variability observed in the unfavorable prognosis group may reflect the overall severity of neurological injury and systemic instability in children with PSH. Severe brain dysfunction may lead to impaired autonomic regulation, resulting in greater fluctuations in vascular tone and blood pressure control. These findings may be related to the limited sample size, and future larger-scale studies are warranted to further validate the prognostic value of the blood pressure coefficient of variation in pediatric patients with PSH. Although studies specifically examining the relationship between PSH itself and blood pressure variability (BPV) are currently scarce, evidence exists in the field of neurocritical care and brain injury that BPV is associated with poor neurological outcomes. For instance, a study utilizing the eICU-CRD database and analyzing patients with traumatic brain injury revealed that systolic blood pressure variability within the first 24 h of admission was significantly associated with an unfavorable prognosis (Zhang et al., 2024). Furthermore, a recent review revealed that during the acute phase of stroke, high BPV is closely linked to unstable cerebral perfusion, increased injury, and adverse outcomes (Rose et al., 2025). On the other hand, children in the unfavorable prognosis group presented with lower GCS scores, indicating that a decreased level of consciousness itself is a significant factor contributing to worse outcomes. These findings are consistent with those of previous studies showing that a severe brain functional impairment is not only is associated with a high-risk background for the development of PSH but is also closely associated with slower recovery, prolonged hospitalization, and poorer neurological functional outcomes (Miao et al., 2023).

In this study, a comparison between children with PSH exhibiting nondipper and reverse-dipper blood pressure rhythm phenotypes revealed no significant differences between the two groups in terms of sex, age, blood pressure variability, or the time from symptom onset to diagnosis. These findings should be interpreted with caution, as the absence of statistically significant differences does not exclude potential variations between different blood pressure rhythm phenotypes, particularly given the limited sample size. However, although the difference did not reach statistical significance, the reverse-dipper group showed a trend toward a higher rate of unfavorable outcomes. While reports on blood pressure rhythm classifications specifically in patients with PSH are scarce in the literature, existing studies emphasize that PSH represents a cyclical state of high autonomic nervous system instability characterized by recurrent paroxysmal surges in sympathetic activity (Meyfroidt et al., 2017). However, blood pressure rhythm phenotypes have also been reported in other disorders and critically ill populations and therefore should not be regarded as specific to PSH. From the perspective of circadian physiology, normal human blood pressure exhibits a diurnal rhythm (a dipping pattern), with a typical nocturnal decrease (a dipping pattern). In contrast, nondipper or reverse-dipper patterns are associated with a sustained predominance of sympathetic activity (Williams et al., 2018; Smolensky et al., 2017). In children with PSH, the reverse-dipper pattern may reflect either a persistently high baseline level of sympathetic activity or impairment of nocturnal inhibitory mechanisms, preventing the autonomic regulatory system from restoring a normal nocturnal blood pressure dip. This rhythm disturbance could be a characteristic manifestation resulting from injury to central control networks, such as the cortical–thalamic–brainstem circuits. When PSH is diagnosed and managed, clinicians’ attention should extend beyond intervening in the sympathetic storms themselves to recognizing abnormal blood pressure rhythm as a marker of overall autonomic dysfunction. Future research should aim to expand the sample sizes, extend follow-up periods, and incorporate other autonomic physiological indicators, such as catecholamine levels and heart rate variability, to further explore the relationships between rhythm types and clinical outcomes.

Although this study revealed important clinical features regarding blood pressure rhythm, blood pressure variability, and hospitalization outcomes of children with severe encephalitis complicated by PSH, several limitations exist. First, this single-center, retrospective study had a relatively limited sample size. Particularly in the subgroup comparisons of blood pressure rhythm phenotypes, the sample size may have been insufficient, and some statistically non-significant findings should therefore be interpreted with caution. These results require further validation in larger, multicenter cohorts. Second, due to the complex etiology and high heterogeneity in the disease course of severe encephalitis, although major clinical variables were compared, potential confounding factors, such as differences in underlying etiology and treatment strategies, may still influence the occurrence and prognosis of PSH. Definitively establishing whether PSH acts as a driver of disease progression or represents a typical manifestation of severe brain injury remains challenging. Furthermore, while the blood pressure data in this study were based on invasive arterial blood pressure monitoring, the correlation between the rhythm classification and the autonomic nervous system status lacked support from direct physiological indicators, such as heart rate variability or catecholamine levels. Moreover, day or night blood pressure differences may be influenced not only by endogenous circadian regulation but also by ICU-related factors, including nursing care, clinical procedures, and the timing of sedation. These factors may attenuate physiological circadian blood pressure variation. This limitation hinders further inference regarding the association between rhythm abnormalities and underlying neurophysiological mechanisms. Future research should involve multicenter, prospective cohort studies with expanded sample sizes to enhance the causal inference regarding the relationships among blood pressure rhythm phenotypes, blood pressure variability, and the prognosis. Additionally, although the PSH-AM has been widely used for PSH identification, the modified version applied in this study was adapted for pediatric patients and has not been formally validated. Therefore, the diagnostic accuracy and generalizability of this modified tool require further prospective validation in larger pediatric cohorts. Last, integrating autonomic nervous system monitoring, neuroimaging, electroencephalography, and metabolic indicators could provide a more comprehensive understanding of the pathophysiological mechanisms of PSH.

## 5 Conclusion

The majority of children with severe encephalitis complicated by PSH exhibited a nondipper or reverse-dipper blood pressure rhythm phenotype. Diastolic blood pressure variability and the GCS score at admission were associated with the prognosis. Compared with those in the control group, the children in the PSH-complicated group were older, experienced prolonged PICU stays, required more mechanical ventilation, and incurred significantly higher hospitalization costs.

## Data Availability

The original contributions presented in the study are included in the article/supplementary material, further inquiries can be directed to the corresponding authors.
